# The complexity of promoting physical activity in English state primary schools: an in-depth qualitative analysis of the role of social context

**DOI:** 10.1186/s12889-025-23471-z

**Published:** 2025-08-25

**Authors:** Robert Walker, Danielle House, Alice Porter, Ruth Salway, Simona Kent-Saisch, Michael Beets, David Revalds Lubans, Frank de Vocht, Russell Jago

**Affiliations:** 1 Population Health Sciences, Bristol Medical School, University of Bristol, Bristol, UK; 2 NIHR Bristol Biomedical Research Centre, University Hospitals Bristol and Weston NHS Foundation Trust and University of Bristol, Bristol, UK; 3 Arnold School of Public Health, University of South Carolina, Columbia, SC USA; 4 Centre for Active Living and Learning, College of Human and Social Futures, University of Newcastle, Callaghan, NSW Australia; 5 Hunter Medical Research Institute, Newcastle, NSW Australia; 6 Faculty of Sport and Health Sciences, University of Jyväskylä, Jyväskylä, Finland; 7 The National Institute for Health and Care Research, Applied Research Collaboration West (NIHR ARC West), University Hospitals Bristol and Weston NHS Foundation Trust and University of Bristol, Bristol, UK

**Keywords:** Physical activity, Children, School-based, Primary schools, Context, Tailored-intervention

## Abstract

**Background:**

Primary schools present an opportunity to equitably promote physical activity. To date, school-based interventions have had limited success. Contextual differences between schools could be a key factor that contributes to this lack of impact. However, the elements that constitute a school’s social context (i.e. the organisational, political, cultural, and sociodemographic characteristics) and how they might impact on primary school physical activity are unclear. The aims of this study were to conduct an in-depth qualitative analysis of state primary school social context related to physical activity and, using this analysis, provide recommendations for policy and practice.

**Methods:**

We conducted one-to-one semi-structured interviews with 33 state primary school staff in Southwest England between November 2023 and January 2024. Staff roles included headteachers/principals (*n* = 5), deputy headteachers/principals (*n* = 5), class teachers (*n* = 7), Physical Education (PE) subject leads (*n* = 8), dedicated PE teachers (*n* = 4), teaching assistants (*n* = 2), and one Parent Teacher Association chair. Staff were recruited from 19 purposively-sampled schools with a range of school sizes and sociodemographic characteristics. We used reflexive thematic analysis.

**Results:**

Five themes were generated that highlighted: (1) the impact of regulatory systems and curriculum pressures created an environment where physical activity was difficult to prioritise; (2) schools with high pupil needs experienced increased pressure, which sometimes impacted staff motivation and wellbeing; (3) senior leadership priorities were key to promoting pupil physical activity, influenced by their personal interests/background/values, regulatory inspections, curriculum pressures, and pupil needs; (4) limited PE training during teacher training impacted teacher confidence that, when combined with curriculum pressures, led many schools to outsource PE to external enterprise; and (5) “passionate” individual staff members who dedicated their own time were important to drive physical activity in the pressured school environment.

**Conclusion:**

Revisions to school regulatory systems and policies are needed to enable schools to prioritise physical activity so it is not dependent on “passionate” individuals. Senior leadership plays a key role in prioritising physical activity in the high pressure state primary school environment. It is important that practitioners and researchers consider these diverse and challenging social contextual factors within English state primary schools in intervention design.

**Supplementary Information:**

The online version contains supplementary material available at 10.1186/s12889-025-23471-z.

## Introduction

Physical activity plays an important role in children’s current and future health and wellbeing [1–3]. The UK Chief Medical Officer [4] and World Health Organization [5] recommend children accumulate an average of one hour of moderate to vigorous intensity physical activity (MVPA) per day across the week. Although physical activity has, on average, returned to pre-pandemic levels in England [6, 7], changes to the way in which children are active after the pandemic has widened certain socioeconomic physical activity inequalities [8–10]. The new physical activity normal in England has been characterised as an increased dependence on structured forms of physical activity, such as active clubs, while the proportion of children from households with lower education attainment who attended community-based active clubs (i.e. a local football club) was down 14% in 2022 compared to pre-pandemic levels [8, 11]. Moreover, the number of children aged 10–11 meeting recommended levels of physical activity remains low at just 41% [7]. It is therefore important that equitable strategies for increasing children’s physical activity levels are developed.

State primary schools (i.e. a publicly funded school that provides free education to children aged 5–11) present an opportunity in which physical activity can be equitably promoted to all pupils [12, 13]. However, most school-based physical activity interventions have been unsuccessful or only yielded small improvements [14–16]. We have recently argued that one potential reason for this is a lack of consideration for the heterogeneity of primary school contexts and the adoption of a “one size fits all” approach to intervention design [17]. Aspects of primary school context comprises its social elements, including organisational, political, cultural, and sociodemographic factors and should be considered on a school-by-school basis so that interventions are appropriate. However, information on which aspects of social context are most influential on physical activity is limited and these have been underreported in primary school-based physical activity intervention literature [18].

In recent years, there has been a shift towards a more tailored approach to school-based physical activity intervention design, which recognises the need for school-specific approaches. For example, Creating Active Schools in the UK [19–21] and the ACTivity PROmotion via Schools (ACTIPROS) ‘toolbox’ in Germany [22] both provide tailored approaches to school-based physical activity. While these approaches take steps towards tailored and bespoke interventions, the role that school social context plays has not been explored in-depth. The aims of this study were to (a) identify key aspects of state primary school social context that appear to influence pupil physical activity, and (b) provide recommendations for policy and practice.

## Methods

### Participants and procedure

This study drew on qualitative data from the Physical Activity via a School Specific PORTfolio (PASSPORT) project [23] collected within two sets of one-to-one semi-structured interviews with state primary school staff. The first set of interviews focused on understanding school context, while the second focused on exploring the feasibility and acceptability of a tailored intervention design. As key data related to context were apparent in both sets of interviews, both datasets were used in the analysis.

In total, 19 state primary schools in the wider Bristol area, England, were purposively sampled to ensure we captured a diverse range of contexts using publicly available local authority and government data [24, 25]. Sampling included a variety of school sizes and geographical locations. Two socioeconomic indicators informed recruitment. Namely, the percentage of pupils in a school eligible for free school meals (FSM) [26], a UK government scheme to provide free meals at school to children from low income families (national average was 23.8% as of 2023 [27]), and school postcode Index of Multiple Deprivation (IMD) decile, a measure of area deprivation, with 10 being least deprived and 1 being most deprived [28]. The percentages of Black, Asian and minority ethnic (BAME) people in the Lower Layer Super Output Area (LSOA, a geographic hierarchy designed to improve the reporting of small area statistics in England and Wales) [24] the school was situated in was also considered to capture diverse cultural and religious school contexts. The national proportion of people who identified as BAME was 18.3% as of 2021 [24].

A total of 33 one-to-one semi-structured interviews were conducted with 32 state primary school staff (one participant participated in both interviews), encompassing headteachers/principals (*n* = 5), deputy headteachers/principals (*n* = 6), class teachers (*n* = 7), Physical Education (PE) subject leads (class teachers who oversee the PE curriculum as a leadership role; *n* = 8), dedicated PE teachers (whose sole role is to teach PE across a school; *n* = 4), Teaching Assistants (TA; *n* = 2), and Parent Teacher Association (PTA) chair (*n* = 1). Sample size was guided by qualitative information power [29]. As such, the research team continuously reflected on and discussed data and recruitment throughout the collection process to determine whether the sample size was adequate and diverse. These reflections were centred on: (1) the aim of the study; (2) participants’ level of specific knowledge and experiences related to our research question; (3) theoretical background of the study; (4) the quality of dialogue; and (5) the adopted cross-case analysis (i.e. our need to include a diverse range of school contexts and participant roles requiring a greater number of participants). Participant demographic and school characteristic information can be seen in Table 1.

**Table 1:** Participant demographic and school characteristic information

**Participant roles**	***n***
Headteacher/Principal	5
Deputy Headteacher/Principal	6
Class Teacher	7
Teaching Assistant	2
Physical Education Lead	8
Dedicated Physical Education Teacher	4
Parent Teacher Association Chair	1
**School characteristics**	
**Urban/rural classifications**	
Urban	10
Suburban	8
Rural	1
**Free School Meal**^1^%	
Below national average (23.8%)	10
Above national average (23.8%)	9
**School postcode Index of Multiple Deprivation** ^**2**^	
1–3	9
4–6	6
7–10	4
**School Lower Layer Super Output Area** ^**3**^ ** Black Asian and Minority Ethnic** ^**4**^ **%**	
Below national average (18.3%)	8
Above national average (18.3%)	10
No data	1
**Number of pupils in school**	
0-200	1
201–400	6
401–600	7
601+	4
No data	1

^1^ a UK government scheme to provide free meals at school to children from low income families

^2^ a measure of area deprivation, with 10 being least deprived and 1 being most deprived

^3^ a geographic hierarchy designed to improve the reportingof small area statistics in England and Wales

^4^ defined as all ethnic groups except white ethnic groups

Interviews were conducted between November 2023 and January 2024 by RW, DH, and AP, and lasted between 22 and 49 min. Six interviews were conducted in person, while the remaining 27 took place via MS Teams. Interviews were audio recorded. The training and experience of interviewers were as follows:

RW has completed postgraduate-level interviewing training and has seven years of qualitative research experience. RW has led qualitative aspects of three interdisciplinary research projects using a variety of methods with both children and adults (> 100 interviews).

DH has specialised in qualitative methods, including interviewing, throughout her career and has conducted interviews in academic and practice-based sectors over the last 12 years. She has trained in interviewing throughout her undergraduate and post-graduate degrees and conducted interviews for circa 10 research projects (academic and practice-based).

AP has completed postgraduate-level interview training through the Bristol Medical School, University of Bristol and Social Research Association, UK. AP has seven years of research experience and has conducted interviews for four research projects (> 60 interviews).

The protocol for this study was published on the Open Science Framework [30]. This study was approved by the University of Bristol, Faculty of Health Science Research Ethics Committee (FREC Ref 15866). Informed consent was obtained for all participants who received a £25 gift voucher as recompense for their time.

### Study materials

Topic guides were developed for both sets of interviews (Supplementary Files 1 and 2) in consultation with a Key Stage 2 (Years 3–6) state primary school teacher. In the first topic guide, we centred questions around opportunities for physical activity in school and the associated “real life” challenges to draw out contextual factors between schools. We felt it was important that participants could define what context means for them in their local, specific context rather than being researcher-defined. Thus, questions were framed so that interviewees were able to define context for themselves, allowing us to understand context from their perspective and ask follow-up questions accordingly. In the second topic guide, questions were framed around initial design ideas for a tailored intervention, including the contextual factors that may lead to implementation challenges.

### Data analysis

This study was based in Critical Realism [31]. Critical Realism provides a meta-perspective that focuses on ontology to avoid an epistemic fallacy, or mistaking empirical reality for reality itself [31]. This perspective provided a focus on mechanisms and structures that may not necessarily exist in the empirical domain of reality. To support this approach, we adopted a reflexive thematic analysis [32] to identify latent and semantic patterns of shared meaning across the data, providing us with a method to move beyond the empirical domain of reality. The analysis was supported by NVivo 13 [33] and involved three stages:

*1) Data familiarisation*

Prior to coding, RW read and re-read interview transcripts to familiarise himself with interview discussions and content before moving onto coding.

*2) Coding*

Codes were developed inductively to capture a range of descriptive, interpretive, and conceptual information of interest, including a range of both semantic and latent codes. To promote reflexivity and nuanced understanding of data, two transcripts for each set of interviews (four in total) were coded independently by RW, DH, and AP who then met to discuss their codes and interpretations of data. The remaining transcripts were then coded twice in full by RW. Coding was iterative to ensure codes were most appropriate and useful in supporting theme generation.

*3) Theme generation*

Themes, which were conceptualised as shared patterns of meaning across data sets centred around a distinct central organising concept, either at a semantic or latent level [32], were generated by RW. This process was split into three phases:

- Generation of initial theme ideas. This began during the coding process. RW made notes, codes, and annotations within the data set related to initial ideas and concepts that warranted further consideration in the broader data and theme generation process.
- Initial theme generation. Initial themes were generated based on extensive familiarisation, coding, and reflexive practice processes. Writing was central to this process, and the clarification of narrative related to our research question.
- Theme refinement. Themes were then refined for clarity, ensuring clear boundaries around their central meaning and organising concept. Data was once again revisited to ensure that important content was not overlooked.

### Qualitative analysis

Five themes were generated related to key aspects of state primary school social context that appeared to influence physical activity. These were: (1) The battle for priorities: primary school pressures in a packed curriculum; (2) It’s not a level playing field: high pupil need can indirectly impact physical activity; (3) It comes through the head: senior leadership shape the school; (4) Jack of all trades: primary school teachers’ lack of training and confidence in PE; and (5) Sundays are for planning PE: The need for passion to drive physical activity in primary schools. Verbatim interview quotes are presented alongside participant role, the percentage of pupils eligible for FSMs in the school, and school IMD decile.

Our analysis distinguishes between two distinct yet related concepts: physical activity and PE. Physical activity was considered as any bodily movement produced by skeletal muscles that requires energy expenditure that can take many forms within school and has many purposes (e.g. PE, active travel to school, active clubs, play at break time) [4]. Whereas, PE is used to more specifically denote the foundational subject taught as part of the English national curriculum, which aims to ensure that all pupils develop competence in a broad range of physical activities, are physically active for a sustained period of time, engage in competitive sports and activities, and lead healthy, active lives [34]. In England, schools are expected to provide a minimum of two hours of PE per week for all pupils, the outcomes of which are standardised across age groups [34]. In the analysis below, PE is used to describe the non-compulsory curriculum-based sessions and physical activity to describe broader opportunities within school that can include PE.

### Theme 1. the battle for priorities: primary school pressures in a packed curriculum

A strong sense of a highly pressured environment and an “almost unachievably busy curriculum” (Participant 14) was expressed in the data. Teachers described significant challenges teaching the breadth of subjects (i.e., Maths, English, music, foreign languages, PE, health and wellbeing). While many felt it important to teach a broad range of subjects, regulatory systems substantially shaped which subjects were deemed ‘more important’ than others and given priority. In England, primary schools are inspected and graded around once every four years by the Office for Standards in Education, Children’s Services and Skills (Ofsted) [35]. Ofsted reports and grades for overall school quality are made publicly available. The impact of these inspections was clear. Participants described a focus on core subjects such as Maths and English which would always be assessed, with foundation subjects (e.g., PE, music, history, and geography), where schools reportedly have some control over which subject would be assessed, becoming less prioritised in the pressured curriculum.

*“The national curriculum is very prescriptive. It’s*,* in my opinion*,* hugely overstuffed. but then we’ve got the pressure of Ofsted*,* and you’ve got external pressure… league tables and that sort of thing. If you don’t have the maths and phonics at Key Stage One*,* then you can’t get the good [Ofsted] grading…If your grading*,* if your judgement is going to be on those things*,* then everything else falls by the wayside of it…” (Participant 1*,* PTA chair; school FSM 37%*,* IMD 2)*.

*“…you don’t really need evidence that they’ve done PE*,* but you need evidence that they’ve finished English… they’re not going to check-up on whether they’ve thrown a ball in PE.” (Participant 4*,* TA; school FSM 48%*,* IMD 1)*.

In comparison to subjects where written assessments are the norm, obtaining tangible outcomes for PE was described as challenging. Subsequently, this further encouraged teachers to focus on subjects with more tangible outcomes that they are required to evidence with senior leadership staff and Ofsted.

*“…so the head says to me*,* when he gave me this [PE Lead] role*,* and I’m all excited about it*,* and I’ve got all these ideas*,* he goes*,* “Oh*,* the thing is*,* Ofsted will never do a deep dive into PE*,* so don’t really worry about it.” (Participant 14*,* PE lead; school FSM 21%*,* IMD 4)*.

### Theme 2. it’s not a level playing field: high pupil need can indirectly impact physical activity

This theme highlights the impact high pupil need can have in the already pressured school environment (Theme 1) and its subsequent, indirect impact on physical activity opportunities. Staff in schools with high levels of pupil need expressed a strong sense of responsibility and requirement to meet the needs of their pupils, many of whom faced high levels of hardship, with some experiencing poverty, having low levels of English, or Special Educational Needs and Disabilities (SEND). This included providing their main meal and school uniform, as well as enrichment and physical activities that pupils would not otherwise have access to. However, meeting an increased requirement for physical activity appeared challenging, as pressured curriculum time (Theme 1) may be further stretched when schools are required to meet highly complex pupil needs. Many schools reported that pupil needs, especially in disadvantaged communities, had increased in recent years, particularly since the COVID-19 pandemic. It was also noted that many children do not meet official criteria of disadvantage that would qualify them, or the school, for additional government financial support. As a result, “disadvantage” may be hidden and school resources to support children with additional needs further stretched.

*“We understand that for some of our children*,* it’s really important they’re active*,* because… the community that we serve*,* some of our children don’t have a garden. They don’t have an outdoor setting… We know that they’re probably going to spend the week at home… It’s trying to build their life chances. Almost level the playing field a little bit. (Participant 11*,* class teacher; school FSM 32%*,* IMD 2)*

*“There are a lot of families who hover on that level of deprivation but sit just above it. They don’t necessarily get benefits*,* they don’t get free school meal entitlement*,* but they probably should.” (Participant 21*,* headteacher; school FSM 10%*,* IMD 3)*.

Participants described the challenges they experienced supporting some children to be physically active. PE was described by some as particularly challenging as it had the potential to cause significant disruption by exacerbating any socio-emotional issues that may stem from challenges the child is experiencing at home. This was also suggested to, at times, impact upon teachers’ energy and wellbeing and led some to avoid activities that can cause disruption.

*“I think behaviour management as well is a massive issue… I know particularly when I was an early career teacher*,* “If I go out and I teach them PE now and it all kicks off*,* is it safer just to stay in the classroom and maybe we’ll do a littler PE lesson because it’s less to handle*,* rather than taking 30 children outside where it could all explode and you could be dealing with more?” (Participant 11*,* class teacher; school FSM 32%*,* IMD 2)*.

*“If you’ve got a tricky class*,* it takes up so much energy. By the end of the day*,* you’re absolutely exhausted*,* and that has major implications on your wellbeing*,* which has implications on wanting to do better…” (Participant 2*,* PE lead; school FSM 32%*,* IMD 2)*.

### Theme 3. it comes through the head: senior leadership shape the school

Participants explained that school priorities were decided by the Senior Leadership Team (SLT). Whilst the regulatory systems and curriculum pressures (Theme 1) and pupil needs (Theme 2) shape their decisions, SLT members bring their own personal values to their role. This includes physical activity, whereby a headteacher who highly values the positive role it could play in their school is more likely to make it a priority in school.

*“It [school priorities] probably does come through me… PE was a subject that I led first*,* so it was in my second year of teaching*,* PE was something I led. And it was something that I always felt passionate about…” (Participant 3*,* headteacher; school FSM 34%*,* IMD 1)*.

Senior leadership support was considered vital to enabling staff to provide physical activity opportunities to pupils. With resources and budgets described as “non-existent” (Participant 10) and “being cut more and more” (Participant 11), senior leadership who valued physical activity were suggested to be more deliberate and considered in their use of budgets and resources to promote sustainable school physical activity. Senior leadership priorities and values extended to decisions that encouraged or enabled staff to provide opportunities in the highly pressured curriculum (Theme 1), for example, use of incentives or policies to encourage staff to volunteer to organise clubs.

*When we take part in competition*,* we have this Nike equipment. So*,* we represent the school*,* we’ve got the shirts*,* we look the business and we’re very proud of our PE curriculum. That couldn’t have happened without money and support from the headteacher. (Participant 12*,* PE lead; school FSM 11%*,* IMD 8)*

*“the senior leadership team*,* working out how we actively encourage our staff to run these clubs. We do offer time back for it. So*,* we say if you run a club for two terms*,* you get a day off in lieu.” (Participant 11*,* class teacher; school FSM 32%*,* IMD 2)*.

### Theme 4. jack of all trades: primary school teachers’ lack of training and confidence in PE

In English primary schools, one teacher teaches most of the subjects. During their teacher training, participants noted a significant lack of PE instruction, with many noting only a few hours of training throughout their course (Theme 1). As a result, teachers’ confidence in PE is lower than for core subjects, such as Maths and English, especially among newly qualified teachers.

*“There’s a lot of discussion and debate in the primary sector about how little teacher training they have on PE*,* maybe a couple of hours during their whole study. So a lot of that would be confidence and skills.” (Participant 15*,* Dedicated PE teacher; school FSM 18%*,* IMD 6)*.

Reflecting this lack of training and confidence, participants described outsourcing some of their PE provision to external, commercial companies. Although this provided some schools with an opportunity for staff to learn from external sport coaches to overcome low levels of training in PE, when combined with the pressures of the national curriculum (Theme 1), these outsourced PE sessions often became time for teachers to catch up on preparation and planning for non-PE curriculum lessons, exacerbating the gap in their PE knowledge and skills. Moreover, it was suggested that external organisations’ PE provision may be taken by sports coaches who may lack pedagogical expertise needed to teach what was considered the broader aspects of PE, such as healthy lifestyle promotion beyond sport. Moreover, sport coaches (who may have good content knowledge) may lack the necessary knowledge and skills to support children’s socioemotional wellbeing and their engagement with the session (Participant 13).

*“This year*,* when teachers are on their PPA [Planning*,* Preparation and Assessment time]*,* so when they’re out of class*,* they have hired in a group of people to come in and deliver not all*,* but a lot of PE lessons for us. Which*,* to me*,* is creating a gap between expert teachers” (Participant 14*,* PE lead; school FSM 21%*,* IMD 4)*.

*“So teachers really love having a sports coach because one*,* they can get a better understanding of that sport and two*,* they can get loads of ideas*,* and three*,* have a bit of respite because they haven’t got to plan that second PE lesson for that whole term.” (Participant 2*,* PE lead; school FSM 32%*,* IMD 2)*.

Stemming from the challenges surrounding teacher training, confidence, and curriculum pressures, some schools made the decision to hire a dedicated PE teacher who was responsible for PE across the school. In contrast to a PE lead, who tends to be a member of teaching staff who has been given the leadership role as part of their wider responsibilities and may or may not be interested in the subject, dedicated PE teachers are able to bring subject expertise and dedicated focus to develop a quality PE curriculum that captures a broad range of sport and healthy lifestyle skills. Yet, the challenges of funding this were apparent, and likely required PE to be highly valued and prioritised by SLT (Theme 3).

*“I’ve been there for eight years as a classroom-based teacher… and you do not have time to do anything like that [developing quality PE lessons] because you’re going to be doing all your marking*,* you’re going to be doing all of your planning*,* you’re going to be doing all of your assessment… mainly Maths and English… So [as a dedicated PE teacher] I’m able to do all of that which they’re not… I’m able to arrange a lot of specialist teaching… but it’s expensive*,* obviously*,* you know.” (Participant 9*,* dedicated PE teacher; school FSM 10%*,* IMD 10)*.

### Theme 5. sundays are for planning PE: the need for passion to drive physical activity in primary schools

In the context of the pressured curriculum and regulatory inspections (Theme 1), meeting pupil needs (Theme 2), SLT priorities (Theme 3), and limited PE training (Theme 4), delivering high-quality PE and physical activity can be very challenging. Participants described a sense of ‘passion’ that was often needed to overcome challenges and drive school physical activity. This passionate person was characterised as an individual who valued physical activity, often having an interest in sport or physical activity in their personal life, and subsequently invested a lot of time and energy into sharing their passion with pupils. Among the schools included in this study, examples of passionate staff members were noted among a wide range of roles, including headteacher/principles, SLT members, class teachers, and PE leads/dedicated PE teachers, who played a key role in embedding physical activity and providing support to other staff.

*“I do generally work on a Sunday as well for two or three hours. So*,* I tend to do classroom stuff in my PPA time. Sundays are for PE… I’m really passionate about sport and PE. So*,* you’ve got to really love PE. The amount of time and effort… the amount that I do outside of school- We do three competitions a week and that runs until 6:00pm.” (Participant 12*,* PE lead; school FSM 11%*,* IMD 8)*.

While passionate individuals played a key role in promoting school physical activity across the whole day, their impact on active extra-curricular club provision was particularly clear. Active clubs that were run voluntarily by staff members, who often shared their passion for a particular sport or activity, were suggested to drive engagement and excitement from the children (Participant 13). However, staff volunteering their time was particularly challenging in an already highly pressured job (Theme 1) that many found exhausting. Subsequently, many schools chose to outsource active clubs to commercial companies in a similar way to PE (Theme 3). Outsourcing active clubs may also create inequalities in terms of access to those from disadvantaged backgrounds due to cost (Theme 2), where externally provided clubs come at a fee to families (unless SLT decided to subsidise with school budgets), whereas teacher-led clubs tended to be free of charge. This may also explain the overall impression in the data that inter-school competitions were decreasing, with passionate staff needed to spend the time required to complete administration (i.e. organising transport/risk assessments) and supervise the children to external venues.

*“My current school doesn’t have any requirements for teachers to run clubs. So we have external parties” (Participant 14*,* PE lead; school FSM 21%*,* IMD 4)*.

*“We’ve got to do a risk assessment for every competition that we do. It takes about an hour to organise every competition and we’re doing three competitions a week every term. If you do the maths*,* it’s a lot of work. My schoolwork*,* because I’m a classroom teacher as well*,* it suffers. But I find that PE is a priority and I enjoy doing it as well.” (Participant 12*,* PE lead; school FSM 11%*,* IMD 8)*.

### Overview of themes

**Fig. 1:**
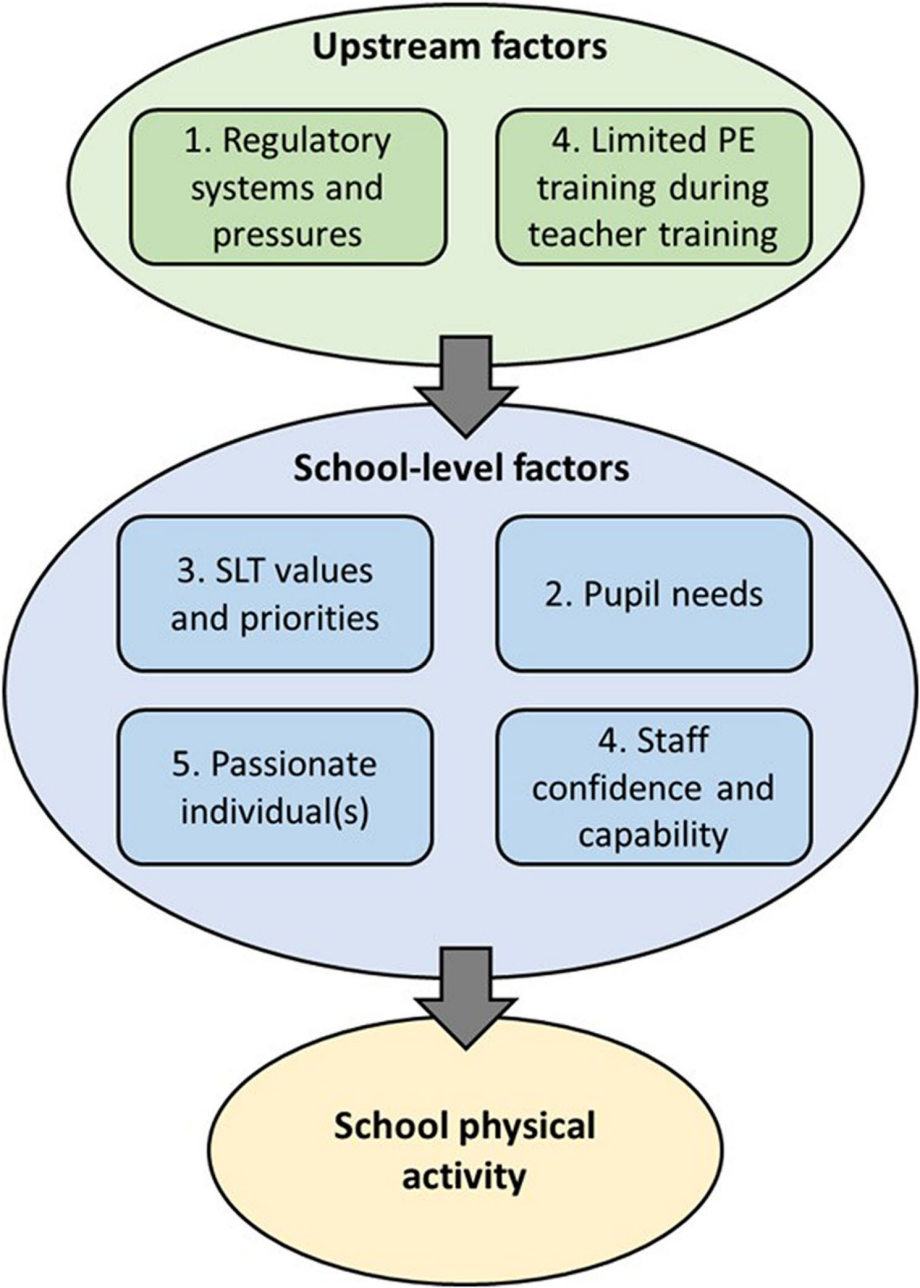
Overview of overarching narrative of key findings

The five themes above outline important aspects of school social context that appear to influence physical activity. Figure 1 presents key findings within these themes and their overall narrative, with numbers indicating the theme they relate to (e.g. the impact of regulatory systems and pressures was discussed in Theme 1). Two upstream factors (i.e. systemic issues beyond the school environment) were identified that were important for understanding school social context. These were: (1) Regulatory systems and pressures and (2) limited PE training during teacher training. Four school-level social contextual factors were identified. These were: (a) SLT personal values and priorities; (b) pupil needs; (c) passionate individual(s); and (d) staff confidence and capability.

## Discussion

Drawing on the perspectives of school staff, this study identified key aspects of English state primary school social context that appeared influential for promoting physical activity. This study adds novel findings that are important in supporting school physical activity promotion.

Aligned with our first theme, the negative impact of regulatory systems and curriculum pressures on school priorities and physical activity have been reported in England [36, 37], Wales [38] and Norway [39]. Competing curriculum and resource demands and PE and physical activity being considered a lower priority than other subjects have been identified as key barriers to physical activity within primary schools [40, 41]. There have been recent calls to ‘upgrade’ PE in English primary schools to a core subject that is regulated and inspected to the same extent as Maths and English [42]. While this would help promote quality of PE, the current “almost unachievably busy curriculum” that we identified presents significant challenges to this. Without coinciding with additional resources, state primary schools would likely not have sufficient capacity to support another core curriculum subject. Perhaps it is for this reason that many countries, including Denmark [43], Canada [44], and Hungary [45], have implemented policies to extend the school day to enable schools to support pupil physical activity. It is therefore possible that such a policy in the UK may need to be considered before PE as a core subject becomes feasible.

Our analysis suggested that the personal values of SLT members and “passionate” staff in promoting school physical activity amongst curriculum and regulatory pressures. This echoes research that suggests staff values, background, experience, and interests, particularly those of the SLT, play a key role in successful intervention implementation and delivering quality physical activity or health interventions in schools [36–39, 46]. This presents a significant issue in the context of stretched school resources, which leaves the extent of health promotion in schools dependent on SLT and staff valuing the activity enough to dedicate limited resources or personal time to. While policy reform would be key to enabling staff to promote health activities, until this, understanding how new and existing physical activity provision can be effectively developed to working within this environment to maximise engagement, feasibility and effectiveness is key.

It is well documented that generalist primary teachers lack confidence to teach PE due to limited opportunities to learn during teacher training [36, 47, 48], which has coincided with a trend towards outsourcing PE to external commercial enterprises [49–52]. Research has highlighted a mixture of views related to this kind of external provision. Positive views include external providers being seen to have greater expertise that allowed pupils to experience a wide range of sports while also providing development and learning opportunities to teaching staff [50–52]. However, criticisms have included that external coaching staff have a tendency to lack pedagogical expertise in PE and may reinforce the notion that teaching PE is equivalent to sports coaching [52], and not just one aspect of the curriculum. Teachers’ perception that external providers hold greater expertise may also lead teachers to engage minimally with the sessions [50], further decreasing their PE skills. In addition, recent research suggested that many teachers want to teach PE but are having less opportunity due to the extent external provision has become prevalent [49]. Our findings echo the discourse surrounding this topic, suggesting that lack of training and confidence remains a key issue in England and may be an influential factor in the decision-making process that is leading more schools to outsource PE, which may further impact staff skills. Our analysis also adds further nuance related to the pressured curriculum and regulatory systems on decisions to outsource PE, with many seeing it as an attractive option that gives additional time to teachers for other, higher-priority activities. It is, therefore, important that policymakers and practitioners consider the impact of both limited PE training and school-level pressures in strategies to enable state primary school teachers to confidently teach PE.

Our findings suggest schools with high pupil need face additional pressures that indirectly impact physical activity. In these cases, other priorities or areas of focus may be needed that may reduce opportunities for physical activity. For example, teachers may lack confidence to adapt PE lessons for SEND pupils [53], which can present its own challenges such as increased planning time [54]. Furthermore, fewer pupils with lower levels of English proficiency meet the expected standard of achievement in primary school than their fluent or native speaking classmates [55], which may lead to schools needing to provide language learning in addition to the busy curriculum. Concerns surrounding the impact of a “tricky” class were also related to increased needs. Reviews within educational research have noted the impact classroom context and student relationships can have on teacher resilience and wellbeing [56, 57], with meta-analysis showing that misbehaviour can lead to teacher emotional exhaustion [58]. Our analysis suggested these challenges also impact teachers’ motivation and willingness for physical activities because of views that interrupting classroom work with physical activity could worsen behaviour. Yet, paradoxically, there is evidence that suggests short physical activity breaks increase children’s on-task behaviour (i.e. the amount of time actively spent in the learning process) [59]. It may therefore be warranted that future interventions are developed to navigate teachers’ concerns related to physical activity being a disruptive activity and how programmes can be adapted to reduce the potential for disruption and meet diverse needs.

We have recently argued for a transition from a “one size fits all” approach to primary school physical activity intervention design towards a more tailored, context-specific approach [17]. The findings of this study provide further evidence for the importance of context-specific approaches, highlighting the influences of sociodemographic, organisational, and individual staff factors and the complexity of delivering physical activity interventions within state primary schools. It is important that current tailored strategies to promote physical activity in state primary schools are designed to fit within context so that interventions can be effective in all schools and not only in schools with individual staff or SLT who value physical activity.

### Implications for policy and practice

Based on the findings of this study, Table 2 outlines four implications for policy and practice. We believe that policy reform is needed to overcome systemic challenges to physical activity promotion, such as revisions to the current format of inspection systems and addressing the lack of PE training during primary school teacher training. Practitioners should also consider how best to encourage SLT buy-in and support schools with high levels of pupil need who may be facing increased pressures.

**Table 2:** Implications for policy and practice

	Key finding	Implication
1	Inspection systems (e.g. Ofsted) and pressured curriculums create challenges to promoting physical activities	Significant policy reform is necessary to enable schools to prioritise physical activity. Currently, these inspection systems are leading to schools focusing their limited resources on core curriculum subjects. By making physical activity a part of these inspection systems schools will be encouraged to improve their provision. However, it is important that this coincides with increased funding and resources to avoid this policy change from becoming an additional pressure in an already highly pressured environment.
2	Senior Leadership Team shape school priorities	Senior Leadership Team buy-in is a key component of promoting physical activity in schools and steps should be taken to encourage and enable senior leaders to improve their school’s physical activity provision. For example, by making physical activity a more focused area of regulatory inspections while providing additional funding. Until systems change, however, greater focus needs to be given to how to work within current systems that make physical activity difficult to prioritise to encourage Senior Leadership Team buy-in.
3	Schools with high pupil needs face increased pressures	Future physical activity promotion in schools must consider how best it can support schools, pupils and staff who may be experiencing greater challenges and how interventions can be tailored to these needs. Specifically, schools within disadvantaged communities may face broader challenges, such as those related to pupil engagement and socioemotional health, that require additional support to ensure that physical activity is equitably promoted.
4	Lack of training and curriculum pressures are encouraging schools to outsource Physical Education	Policy reform to ease school pressures and provide more Physical Education training to teachers before they are fully qualified is needed to ensure teachers feel supported and confident to teach Physical Education. This will allow teachers to confidently teach quality Physical Education and alleviate the costs associated with outsourcing these aspects of the curriculum to external commercial organizations.

### Strengths and limitations

Our study has two key strengths. Firstly, through purposive sampling, we were able to recruit participants employed in a range of roles from schools in socioeconomically, geographically, and ethnically diverse areas, which gave our data a broad and balanced mix of perspectives. However, this study was not without limitations. Primarily, it was evident in the data that school context is highly complex and has the potential to change over time. While the one-to-one semi-structured interview method used provided key insights, there exists a need to go beyond the semi-structured interview format into further, in-depth work to fully experience and encapsulate school context and how key aspects interact on a day-to-day basis. Secondly, despite carefully planned recruitment processes, the voluntary nature of this study meant that it is possible that less active schools did not participate due to feelings of not wanting to be placed under scrutiny. This may have led to an imbalance of more physical activity-orientated schools participating in this study. Nevertheless, physical activity levels in England have been linked to both socioeconomic status and ethnicity [60], and our successful recruitment of these schools may have helped to alleviate this limitation.

## Conclusion

This study has highlighted the importance of considering school social context when designing physical activity interventions. Regulatory systems and curriculum pressures made physical activity difficult to prioritise, which may be even greater in schools with high pupil need. Senior leadership priorities, which were influenced by their personal values, pupil needs, and regulatory inspections, were key to physical activity. The lack of confidence to deliver PE among teachers due to limited PE training, combined with curriculum pressures, encouraged many to outsource PE, which may exacerbate teachers’ gaps in knowledge and miss some key educational aspects of the PE curriculum. “Passionate” individual staff members were important to drive physical activity in the pressured school environment through dedicating their own time. We suggest that revisions to regulatory systems and policies are needed to enable schools to give stretched curriculum time to physical activity. It is also vital that practitioners and researchers consider the implications of diverse school context in future intervention design to ensure that they are feasible and effective across schools.

## Supplementary Information

Supplementary Material 1.

Supplementary Material 2.

## Data Availability

As the PASSPORT project is still ongoing, data are not currently available. At the end of the project, data will be published as a restricted access dataset on the University of Bristol’s data repository (https://data.bris.ac.uk/data/) and access granted to approved researchers on request.
